# COVID‐19–Induced Narcolepsy Type I With Cataplexy: A Short Report

**DOI:** 10.1111/jsr.70372

**Published:** 2026-05-29

**Authors:** Torsten Vinding Merinder, Martin Langeskov‐Christensen, Filip Kirov

**Affiliations:** ^1^ Department of Neurology Viborg Regional Hospital Viborg Denmark; ^2^ Department of Clinical Neurophysiology Aarhus University Hospital Aarhus Denmark; ^3^ Department of Clinical Medicine Aarhus University Aarhus Denmark

**Keywords:** COVID‐19, excessive daytime sleepiness, hypnagogic hallucinations, hypocretin deficiency, post‐viral autoimmunity, sleep–wake regulation

## Abstract

Narcolepsy type I is a rare neurological sleep disorder characterised by chronic excessive daytime sleepiness, cataplexy, and low orexin (hypocretin) levels. Infection with SARS‐CoV‐2 has been linked to neurological and neuropsychiatric sequelae, but cases of post‐COVID narcolepsy remain extremely uncommon. We report a previously healthy 42‐year‐old Caucasian female who developed excessive daytime sleepiness, cataplexy, and hypnagogic/hypnopompic hallucinations within 3 months of confirmed SARS‐CoV‐2 infection. Polysomnography (PSG) showed disturbed nocturnal sleep with a high arousal index and periodic limb movements and mild sleep apnea, while the multiple sleep latency test (MSLT) showed a mean latency of 1 min with sleep‐onset rapid eye movement (REM) periods in all naps. Cerebrospinal fluid hypocretin was markedly reduced (< 40 ng/L), confirming narcolepsy type I. Following the diagnosis, medical treatment was initiated—with methylphenidate for hypersomnolence followed by venlafaxine for cataplexy, which led to a substantial symptomatic improvement, enabling a return to daily functioning. The case contributes to the minimal literature on narcolepsy following COVID‐19 and supports hypotheses of autoimmune‐mediated destruction of hypocretin‐producing neurons triggered by viral infection. Clinicians should maintain a high index of suspicion for primary sleep disorders in patients with persistent fatigue and hypersomnolence after COVID‐19, as early recognition and targeted treatment markedly improve outcomes.

## 1 Introduction

Narcolepsy is a chronic neurological sleep disorder presenting with disturbed sleep–wake regulation, typically characterised by excessive daytime sleepiness (EDS) and sleep attacks. In type I cases, this includes cataplexy and reduced cerebrospinal fluid hypocretin concentrations (American Academy of Sleep Medicine 2023; Scammell 2015; Dauvilliers et al. 2007). The prevalence of narcolepsy is estimated to be 25–50 per 100,000 individuals (Scammell 2015; Dauvilliers et al. 2007) with an estimated incidence of 1 per 100.000 individuals. The selective destruction of hypocretin‐producing neurons in the lateral hypothalamus is suspected to be a target of autoimmune processes, as recent emerging evidence has implied; however, the precise aetiology remains incompletely understood (Garrido‐Suárez et al. 2022; Kornum et al. 2011; Liblau et al. 2015).

An infection with SARS‐CoV‐2 has been attributed to a wide range of both acute, post‐acute, or chronic neurological manifestations, including encephalitis, cerebrovascular disease, peripheral neuropathies, and post‐viral fatigue syndromes (Ellul et al. 2020; Taquet et al. 2021). COVID‐19 may act as a trigger for autoimmune‐mediated disorders, and rare cases of narcolepsy have been reported following both infection and vaccination (Moura et al. 2022; Künstler et al. 2025; Pizza et al. 2014). We report a case of narcolepsy type I with cataplexy in a previously healthy adult following a documented COVID‐19 infection. To our knowledge, only two similar cases have been reported worldwide (Moura et al. 2022; Künstler et al. 2025), making this an important contribution to the growing literature on post‐COVID neurological sequelae and syndromes.

## 2 Methods

The case report outlines symptom onset and the diagnostic and treatment course. Data were collected at Viborg Regional Hospital, with PSG (polysomnography) and MSLT (multiple sleep latency test) performed at the Department of Neurophysiology, Aarhus University Hospital. Informed consent was obtained, and all procedures complied with applicable regulations and ethical standards.

## 3 Case Presentation

The patient was a previously healthy 42‐year‐old Caucasian female with no psychiatric, cardiac, immunopathological or neurological history and no known antecedents with neurological or sleep‐related disorders. In May 2022, she was admitted to Aalborg University Hospital following recurrent episodes of sudden loss of muscle tone.

### 3.1 History and Symptomatology

The patient was referred to the acute ward by her general practitioner in May 2022 following the development of progressive weakness in the legs, arms, hands, and neck (Figure 1). Her clinical history regarding infections included two PCR‐confirmed episodes of SARS‐CoV‐2 infection: the first during pregnancy in March 2020, and the second in January 2022. She was unvaccinated at the time of the first infection, during which she experienced fever, myalgia, and pharyngitis. Following childbirth, she received two doses of the Pfizer‐BioNTech vaccine: in November 2021 and in December 2021. She contracted a second COVID‐19 infection in late January 2022. The clinical course was milder, presenting with cold‐like symptoms without fever; however, in the months following January 2022 she had gradual development of prodromal symptoms in the form of muscle weakness, heaviness in the lower extremities and a sensation of ‘jelly‐like’ legs. She experienced episodic exacerbation, and her legs occasionally gave way. During April‐early May 2022, and concomitant with increasing general fatigue, the weakness progressed to her upper extremities and neck. Episodes were brief but frequent (several per hour), often accompanied by mild tremor, blurred vision, and impaired hand coordination, leading her to stop driving.

**FIGURE 1:**
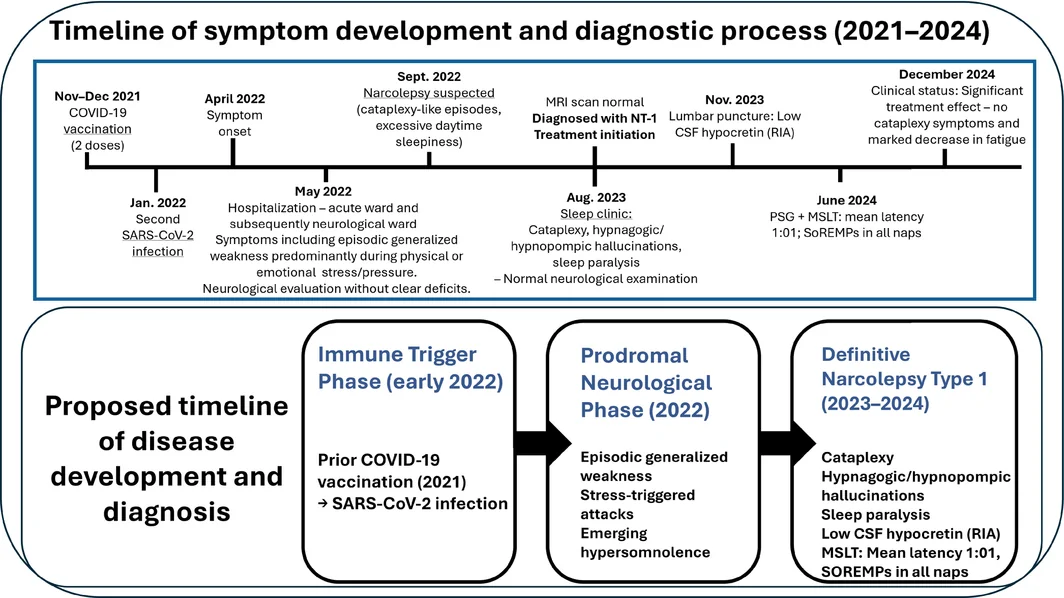
Case timeline. Overview with primary findings, examinations and results.

During her subsequent admission in May of 2022, hospital staff observed multiple episodes of extremity loss of muscle tone followed by tremor and neck twitching, with loss of postural control and a substantial risk of falls. Examples include an observed task situation where the patient attempts to make coffee. She strains to reach the cabinet and retrieve the coffee container, opening it easily. While pouring coffee into the filter, she develops tremulousness and spills coffee onto the table, after which she experiences an episode of weakness, requiring her to sit down. After a brief pause, she completes the remaining activity. She completes the task with marked effort and increased time usage, reporting significant fatigue, and being visibly emotionally affected.

The neurological examination showed bilateral upper limb weakness with preserved osteotendinous reflexes, cranial nerve function and sensation, and no ataxia was noted. Simpson's test was positive. Neurological examinations were not performed specifically during episodes. Brain computed tomography (CT) was normal, and other paraclinical tests were unremarkable. She was referred to Aalborg University Hospital for further neurological evaluation. Myopathy and neuromuscular transmission disorders were initially considered but later excluded. The neurological examination revealed no clear deficits, and the patient was discharged with no underlying organic cause suspected, likely reflecting an initially unclear clinical presentation not suggestive of a sleep disorder. The patient was later referred to our sleep clinic due to persistent symptoms.

### 3.2 Sleep‐Related Symptoms and Clinical Evaluation

Approximately 3 months after the second SARS‐CoV‐2 infection, in April 2022, the patient developed excessive daytime sleepiness. Upon subsequent evaluation at the Department of Neurology's Clinic for Sleep Medicine at Viborg Regional Hospital, in August 2023, the patient reported typical cataplexy with brief episodes (30 s) of sudden muscle tone loss, primarily involving the knees and axial musculature. The episodes were predominantly triggered by physical exertion or emotional stimuli (especially laughter or worry), with preserved consciousness throughout. The patient described fragmented sleep, and hypnagogic and hypnopompic hallucinations, with vivid dream‐like experiences at sleep onset and upon awakening, including continued dream imagery despite awareness of being awake. She reported episodes of sleep paralysis in the morning, with preserved awareness and a transient inability to move. The neurological examination was normal, and her BMI was 30.1, consistent with grade I obesity, prior to the PSG study. Blood pressure in 2022 was 104/81 mmHg and subsequently found to be unremarkable during follow‐up. Her self‐reported Epworth Sleepiness Scale (ESS) score was 22 (Figure 2), with an interviewer‐validated score of 15. The Ullanlinna Narcolepsy Scale score was 39/44.

**FIGURE 2:**
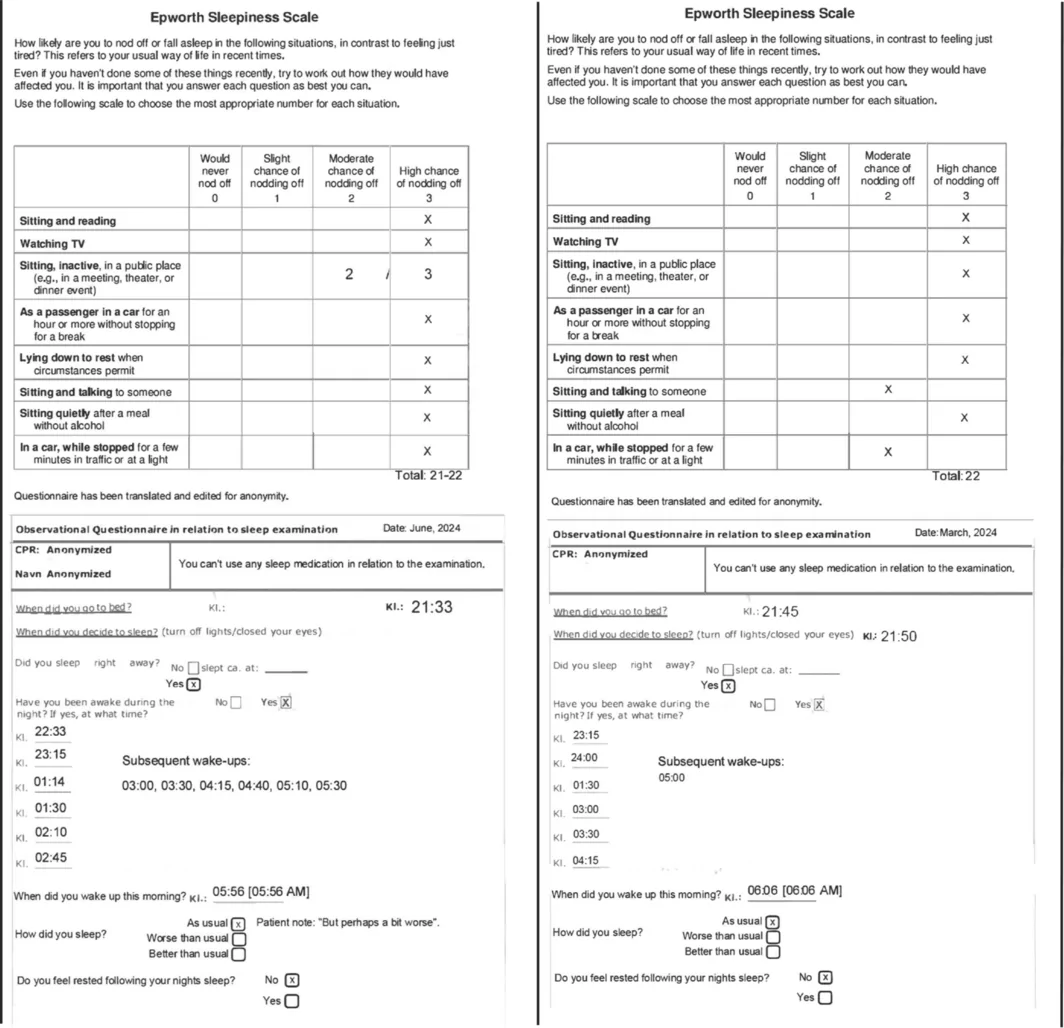
Epworth (March and June 2023). The self‐reported questionnaire details both significant quantitative and qualitative sleep disturbances influencing daytime sleepiness.

## 4 Results

The patient underwent PSG and MSLT on two occasions, as the initial PSG was inconclusive due to a technical issue. A subsequent PSG in June 2024 (Table 1, Figure 3) revealed impacted nocturnal sleep quality, an elevated arousal index, periodic limb movements, and mild obstructive sleep apnea (OSA) not requiring intervention, with an apnea‐hypopnea index (AHI) of 7.4. Sleep fragmentation (Figure 3) may reflect snoring, respiratory effort–related arousals (RERAs), and obstructive events with desaturation. PSG suggested a positional component, with fewer events and arousals and N3 sleep in the left lateral decubitus position. A potential association between increased periodic limb movements and recurrent respiratory‐related arousals could also be considered.

**TABLE| 1:** PSG and MSLT results (June 2024).

PSG findings	
Variable	Result
Study examination parameters	
Total recording time (TRT) (min)	503
Total sleep time (TST) (min)	340.5
Wake after sleep onset (min)	162.5
Latency and efficiency	
Sleep latency (min)	4
REM latency (min)	56
Sleep efficiency (%)	67.7
Staging and distribution	
N1 (% TST)	36.6
N2 (% TST)	31.4
N3 (% TST)	20.3
REM (% of TST)	11.7
Arousal metrics	
Arousal index (/h)	49.5
Spontaneous arousals (/h)	44.1
Respiratory arousals (/h)	0.7
RERAs (/h)	0
Respiratory and motor parameters	
Apnea‐hypopnea index total (/h)	7.4
Apnea‐hypopnea index supine (/h)	11.8
Oxygen desaturation index, total (/h)	16.7
Oxygen desaturation index supine (/h)	26.1
Mean SpO2 (%)	90.7
PLMS index (/h)	36.7

MSLT findings			
Nap trials	Sleep latency (min)	SOREMP	REM onset
Nap 1	2 min 2 s	Yes	2 min, 23 s
Nap 2	17 s	Yes	2 min, 23 s
Nap 3	1 min 17 s	Yes	3 min, 31 s
Nap 4	18 s	Yes	2 min, 26 s
Nap 5	1 min 10 s	Yes	4 min, 19 s
Mean sleep latency	1 min 1 s		
Staging	Sleep in stage 1 and 2		
EEG/ECG	No abnormal findings		

*Note:* The abnormal findings during sleep examination support the hypothesis of narcolepsy, with shortened mean sleep latency and 5 total SOREMPs (present in all recordings).

Abbreviations: ECG, electrocardiogram; EEG, electroencephalography.

**FIGURE 3:**
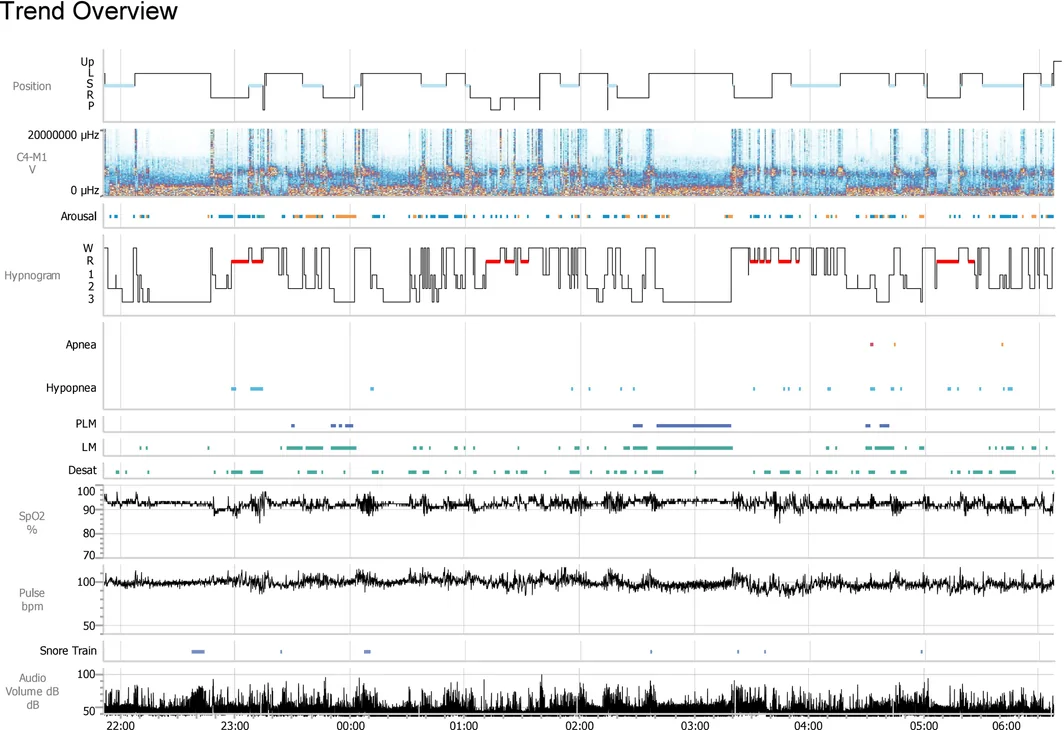
Secondary Polysomnography (June 2024) in trend overview. The examination reveals reduced nocturnal sleep quality, an elevated arousal index, periodic limb movements and mild sleep apnea with an apnea‐hypopnea index of 7.4.

The MSLT demonstrated a mean sleep latency of approximately 1 min, with sleep‐onset REM periods in all naps. Cerebrospinal fluid analysis using radioimmunoassay (RIA) demonstrated a severely reduced hypocretin‐1 concentration below 40 ng/L (Figure 4). Cerebral MRI performed in August 2023 was normal without hypothalamic injury, and routine biochemical analyses were normal. Together, criteria for narcolepsy type I with cataplexy were met, with a comorbidity of mild–moderate OSA accompanied by grade I obesity (American Academy of Sleep Medicine 2023; Scammell 2015; Dauvilliers et al. 2007).

**FIGURE 4:**
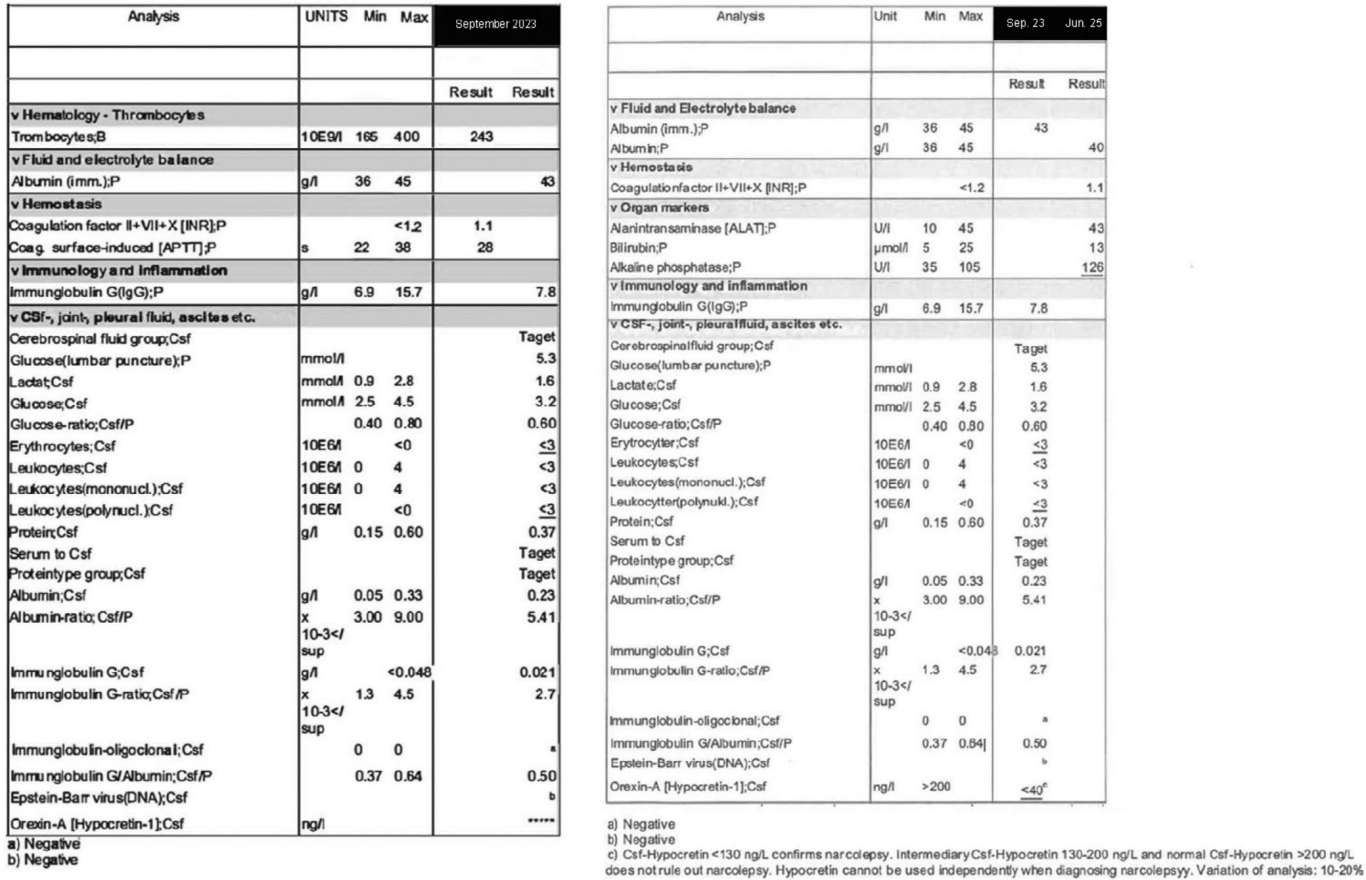
Lumbar puncture findings and biochemical data (Sept. 2023, June 2025). The general results of September 2023 are unremarkable and within standard parameters. The lumbar puncture reveals a severely reduced hypocretin level of below 40 ng/L (standard level above 200 ng/L). Hypocretin‐1 in cerebrspinal fluid was measured using radioimmunoassay (RIA).

### 4.1 Treatment and Follow‐Up

Following the diagnosis, the primary focus of medical treatment was to alleviate cataplexy and improve wakefulness. The patient initiated methylphenidate to enhance wakefulness and subsequently venlafaxine, which reduced cataplexy. Her average frequency of cataplexic episodes was estimated at 10–15 daily. After initiation of modafinil (300 mg daily, with an additional 100 mg as needed), methylphenidate (10 mg twice daily and 10 mg as needed), and venlafaxine (37.5 mg daily), the frequency of cataplexy decreased significantly to approximately 20 cataplexic episodes per month, with a brief increase in relation to job initiation. At follow‐up, her self‐reported ESS score had improved to 13–14. Over several months, she regained functional capacity and daily energy, resumed social and occupational activities, and reported marked improvement in her daily energy, and she is currently engaged in a graded job training program.

## 5 Discussion

Narcolepsy type I (NT1) is defined by cataplexy and low cerebrospinal hypocretin levels, whereas type II (NT2) lacks cataplexy with generally preserved hypocretin signalling (Scammell 2015; Dauvilliers et al. 2007). The distinction is clinically important, as NT1 typically presents with more pronounced daytime sleepiness, emotional triggers for cataplexy, and greater functional impairment. Infections such as influenza A and H1N1 vaccination have been identified as possible triggers for NT1, suggesting an autoimmune mechanism targeting hypocretin‐producing neurons in genetically predisposed individuals (Kornum et al. 2011; Pizza et al. 2014; Mahlios et al. 2013; Partinen et al. 2014; Kornum and Jennum 2020). Although neurological complications of COVID‐19 are increasingly recognised, cases of NT1 following SARS‐CoV‐2 infection remain exceedingly rare (Moura et al. 2022; Künstler et al. 2025). Furthermore, the characteristics of the case, the patient demographic, the timeline of COVID‐19 infection and the known rarity of vaccination‐related sleep disorders (Jeong et al. 2025) undermine the plausibility of the sleep disorder being vaccine‐associated and strengthen the post‐infectious pathophysiological possibility.

Our case demonstrates an initially non‐classical clinical presentation evolving into a fully developed classical NT1 phenotype following COVID‐19 infection, supporting the hypothesis of possible post‐viral initiation of sleep disorders such as narcolepsy. This emphasises the need for clinicians within all fields, particularly neurology and infectious diseases, to be alert for primary sleep disorders in patients presenting persistent hypersomnolence or cataplexy following viral infections including COVID‐19. Heightened awareness, early recognition, and timely referral to sleep specialists can improve quality of life through targeted pharmacological and behavioural interventions and reduce diagnostic delay, thereby enhancing self‐management. As illustrated, narcolepsy can substantially impair daily functioning, underscoring the need for prompt intervention.

Limitations of this case report include its limited generalizability, lack of genetic or immunological profiling, and the inherent inability to establish a direct causal link between SARS‐CoV‐2 infection and NT1. Moreover, testing for Influenza A/B was not performed during the second infection; therefore, the possibility of a concomitant mixed infection, acting as a confounder, following symptom debut cannot be excluded. We have considered HLA class II determination in this case. However, given that the patient's clinical presentation is consistent with narcolepsy type I, supported by low cerebrospinal fluid orexin levels on radioimmunoassay analysis with pathological findings on PSG and MSLT, we believe that these findings, when taken together, support the diagnosis of narcolepsy type I. Although causality cannot be established through direct inference, the close temporal link to SARS‐CoV‐2 infection, thorough diagnostic work‐up, and exclusion of alternative causes support a post‐infectious, possibly autoimmune‐mediated induction of NT1.

To improve early recognition, intervention, and our understanding of pathophysiological mechanisms and narcolepsy as a phenotype, further research is warranted. Future research directions could elaborate on promising emerging areas, such as longitudinal cohort studies exploring post‐viral autoimmune phenomena, genetic susceptibility factors such as HLA‐DQB1*06:02, and biomarkers of neuroinflammation, as well as the general incidence of sleep disorders following viral infections.

## 6 Conclusions

COVID‐19 infection may precipitate and possibly initiate narcolepsy type I in predisposed individuals, as reported in this case of NT1 post‐SARS‐COVID‐19 infection comorbid with mild–moderate OSA. We believe it would be relevant for clinicians to maintain a high index of suspicion when they are faced with patients presenting symptoms of hypersomnolence, cataplexy, and disrupted nocturnal sleep in the months following SARS‐CoV‐2 infection. Early recognition and treatment significantly improve patient outcomes. Further studies are warranted to elucidate the immunological mechanisms underlying post‐COVID narcolepsy and the secondary development of sleep disorders.

## Author Contributions

**Torsten Vinding Merinder:** writing – original draft, writing – review and editing, conceptualisation. **Martin Langeskov‐Christensen:** writing – review and editing. **Filip Kirov:** writing – review and editing including analysis and interpretation, supervision, conceptualisation.

## Funding

The authors have nothing to report.

## Ethics Statement

Ethics approval (dated September 2025) has been submitted separately.

## Consent

Written patient consent was obtained, and no unattributed external material is included.

## Conflicts of Interest

The authors declare no conflicts of interest.

## Supporting information


**Data S1:** Supporting Information.

## Data Availability

The data that support the findings of this study are available from the corresponding author upon reasonable request.
