# Supplementary material for: COVID‐19–Induced Narcolepsy Type I With Cataplexy: A Short Report
Source: J Sleep Res. 2026 May 29;35(5):e70372. doi: 10.1111/jsr.70372 (PMC13619020; doi:10.1111/jsr.70372)
Supplement: Supplementary file 1 [file JSR-35-e70372-s001.docx]

# **Statement regarding other qualitative and supplementary data**

During diagnostic evaluation, the patient completed a general questionnaire, the Pittsburgh Sleep Quality Index, the Cambridge-Hopkins Restless Legs Questionnaire, the Ullanlinna Narcolepsy Scale, as well as a sleep diary with self-reported sleep monitoring during 10 days of sleep at home, which are available upon request. Similar findings to those reported in these self-evaluation tools are detailed in the Epworth questionnaires, and in the reports of the PSG and MSLT. The full report of the initial and secondary examinations using PSG and MSLT, as well as the report of the MRI-scan, is available upon request.
